# The Role of Glial Cells in Synaptic Dysfunction: Insights into Alzheimer's Disease Mechanisms

**DOI:** 10.14336/AD.2023.0718

**Published:** 2024-04-01

**Authors:** Yang Yu, Ran Chen, Kaiyue Mao, Maoyan Deng, Zhigang Li

**Affiliations:** ^1^Scientific Research Center, The Seventh Affiliated Hospital, Sun Yat-sen University, Shenzhen, China.; ^2^School of Medicine, Sun Yat-sen University, Shenzhen, China.; ^3^Shenzhen Key Laboratory of Chinese Medicine Active Substance Screening and Translational Research, Shenzhen, China.

**Keywords:** Microglia, Astrocyte, Oligodendrocyte, Oligodendrocyte precursor cell, Synaptic dysfunction, Alzheimer’s disease

## Abstract

Alzheimer's disease (AD) is a devastating neurodegenerative disorder that impacts a substantial number of individuals globally. Despite its widespread prevalence, there is currently no cure for AD. It is widely acknowledged that normal synaptic function holds a key role in memory, cognitive abilities, and the interneuronal transfer of information. As AD advances, symptoms including synaptic impairment, decreased synaptic density, and cognitive decline become increasingly noticeable. The importance of glial cells in the formation of synapses, the growth of neurons, brain maturation, and safeguarding the microenvironment of the central nervous system is well recognized. However, during AD progression, overactive glial cells can cause synaptic dysfunction, neuronal death, and abnormal neuroinflammation. Both neuroinflammation and synaptic dysfunction are present in the early stages of AD. Therefore, focusing on the changes in glia-synapse communication could provide insights into the mechanisms behind AD. In this review, we aim to provide a summary of the role of various glial cells, including microglia, astrocytes, oligodendrocytes, and oligodendrocyte precursor cells, in regulating synaptic dysfunction. This may offer a new perspective on investigating the underlying mechanisms of AD.

Alzheimer's disease (AD), the most common form of dementia, is a progressive neurodegenerative disorder that causes cognitive dysfunction, language difficulties, social impairments, and behavioral challenges [1]. AD is primarily diagnosed through behavior and neuro-psychological tests, as there are currently no biomarkers for early detection. The majority of AD patients are over the age of 65, with 95% showing symptoms at this age [2]. Despite being recognized for over 100 years, the causes of AD remain largely unknown [3]. In the brain, two key pathologies associated with AD are extracellular senile plaques made of amyloid-β (Aβ) and intracellular neurofibrillary tangles made of Tau protein. The Amyloid cascade hypothesis and the Tau hypothesis are the most widely accepted and supported theories of AD. Despite numerous drugs being tested in animal models and phase II/III clinical trials, most have failed to produce positive results [4]. Thus, it is crucial to continue exploring the mechanisms behind AD and uncovering altered signaling pathways, molecules, or activities. Recently, many researchers have shed light on the importance of glial cells and their functions in both healthy brain activity and disease development.

Approximately 100 million glial cells are present in the healthy adult human brain. The vital functions of glia in the central nervous system have been widely studied, including supplying nutrition to neurons, contributing to their development by surrounding them, and acting as cleaners to eliminate pathogens and dead neurons. Recent advancements in methodology using the isotropic fractionator counting method have shown that the ratio of glial cells to neurons in the human brain is approximately 1:1, rather than 10:1 as previously believed with traditional counting methods [5]. This ratio remains constant across various stages of brain development and in healthy brains. It is known that glia, including astrocytes, oligodendrocytes, and microglia, play a significant role in the development, growth, maturation, and activity of neurons. Their numbers increase alongside the increase of neurons (axons) [6], highlighting the significance of the interaction between glia and neurons. Further understanding of the communication and signaling pathways between glia and neurons may reveal the underlying mechanisms of AD.

Loss of synapses has been observed in the early stages of AD and mild cognitive impairment [7]. A synapse is a junction between neurons or between a neuron and its target cell. It can be classified into two types: chemical synapses and electrical synapses, each with distinct functions and structures. In the human brain, chemical synapses are the dominant type. They consist of a presynaptic part, a postsynaptic part, and a 20 nm synaptic cleft [8]. Synapses transmit signals and materials between neurons in a unidirectional manner. Neurotransmitters, which can be molecules or neuropeptides, are loaded into synaptic vesicles (around 40 nm in diameter) on the presynaptic side and released by the synaptic vesicle cycle and exocytosis. Then, they interact with receptors on the postsynaptic membrane and activate or deactivate ion channels. Generally, either a fast ligand-gated ion channel or a slow G-protein receptor is activated after binding [9]. Some small molecule chemicals, but not neuropeptides, can next be re-uptaken by glia, with studies indicating that astrocytes play a significant role in glutamate clearance [10].

Classifying synapses can be done in various ways based on their morphology, function, and activity. Two commonly accepted methods are: 1) by neurotransmitter type released from the presynapse, including acetylcholine, GABA, dopamine, adrenaline, and others [9]. 2) by effect on the postsynaptic membrane, categorized as excitatory, inhibitory, or non-channel synapses [11]. Of these, excitatory and inhibitory synapses are the most prevalent and significant in the brains. The balance between excitatory and inhibitory synapses is crucial to brain function. A study showed that the ratio of excitatory to inhibitory synapses is higher in the parietal cortex of early-onset AD patients, but not in other forms of dementia [12].

Studies have shown that the number of synapses in the brains of AD patients is significantly lower compared to healthy controls [13]. Synaptic dysfunction and loss are linked to cognitive impairment and AD progression. This occurs earlier than the formation of amyloid plaques and neurofibrillary tangles [14]. For example, a study with P301S mutant tau transgenic mice showed neurofibrillary tangles appearing at 6 months, while synaptic pathology had already started at 3 months [15]. Multiple studies on different AD transgenic animal models have found clear synaptic dysregulation and deletions, accompanied by learning and memory deficits [16, 17]. The recovery of cognitive function has been demonstrated by altering the excitatory synaptic transmission dysfunction in the hippocampus using antibodies [18] and inhibitors [19], suggesting that targeting synaptic dysfunction may be a new therapy for AD. The role of Aβ and Tau in synaptic dysfunction has been studied [20], but the mechanisms behind glia-induced synaptic dysfunction remain largely unknown.

Overall, synaptic dysfunction in AD occurs early and contributes to cognitive disorders in patients. This review summarizes the role of glia in inducing synaptic alterations and dysfunction in AD. The functions and dysfunctions of synapses and interactions between glia and synapses will be discussed in more detail below.

## 1. Glia-synapse communication in AD

### 1.1. Neuroinflammation and its effect on synaptic function

Neuroinflammation is commonly defined as the inflammatory response occurring within the central nervous system (CNS), playing a pivotal role in the pathogenesis of neurodegenerative diseases. We acknowledge that microglia and astrocytes are the principal participants in neuroinflammation. The impact of neuroinflammation on synaptic function is multifaceted, encompassing various aspects. Emerging studies suggest that neuroinflammation impairs neuronal development and maturation, thereby influencing synaptic plasticity. In the context of an inflammatory response, the generation of inflammatory mediators, including pro-inflammatory cytokines (such as IL-1β, TNF), chemokines (such as CX3CL1), as well as small molecular messengers like nitric oxide (NO) and reactive oxygen species (ROS), exerts direct effects on neuronal excitatory and inhibitory regulation. These mediators can disrupt synaptic signaling, perturb synaptic connections, induce neuronal degeneration and apoptosis, and ultimately contribute to cognitive impairment [21-25].

### 1.2. Glial involvement in synapse loss and synaptic density changes

As mentioned previously, glial cells exert a significant influence on the maintenance of both structural integrity and functional efficiency within the nervous system. In the context of neurodegenerative diseases, dysfunction of glial cells results in the depletion of synapses and disturbances in synaptic plasticity. Glial cells actively engage in the clearance of neurotransmitters and other debris from the synaptic cleft, thereby ensuring the proper transmission of signals between neurons [26]. Additionally, they release regulatory substances, such as glutamate transporters, which aid in maintaining a balanced concentration of glutamate between neurons. This regulation, in turn, governs excitatory and inhibitory activities. Furthermore, glial cells possess the ability to synthesize and release neurotrophic factors such as brain-derived neurotrophic factor (BDNF) and nerve growth factor, which are integral in promoting neuronal survival and facilitating synapse formation [27, 28]. Moreover, in instances of neuroinflammation and injury, glial cells can become activated and release inflammatory cytokines, thereby participating in neuroimmune responses and influencing synaptic function [29]. Therefore, glial cells have the ability to regulate synapse loss and changes in synaptic density. However, the intricate nature of synaptic loss involves multiple factors and interactions among diverse glial cell types. Consequently, the precise role of glial cells in this process remains predominantly unexplored.

### 1.3. Impact of glial dysfunction on cognitive decline in AD

Glial cells play a crucial role in mediating synapse loss and alterations in synaptic plasticity. Previous studies have demonstrated that aberrant activity and dysfunction of glial cells in AD are associated with cognitive decline. Pathological examinations of AD brains reveal an abundance of activated microglia and astrocytes surrounding senile plaques [30, 31]. Enhanced microglial phagocytosis and Aβ clearance mediated by microglia have been shown to alleviate cognitive impairment in AD mouse models [32]. Recent investigations have highlighted that astrocyte TDP-43 dysfunction contributes to cognitive impairment through abnormal chemokine-mediated interactions between astrocytes and neurons [33]. Furthermore, other glial cell types, including oligodendrocytes and oligodendrocyte precursor cells (OPC), regulate neurotransmitter and interneuron signal transmission, thereby influencing cognitive function. Dysfunction of glial cells can lead to cognitive decline and plays a significant role in the pathogenesis and progression of AD. However, due to the complex pathology of AD, further research is necessary to elucidate the precise involvement of glial cells in this disease. This review provides a comprehensive overview of the role and underlying mechanisms of glial cell-mediated synaptic dysfunction in AD, along with relevant preclinical and clinical studies.

## 2. Microglia and synaptic dysfunction

**Figure 1.**
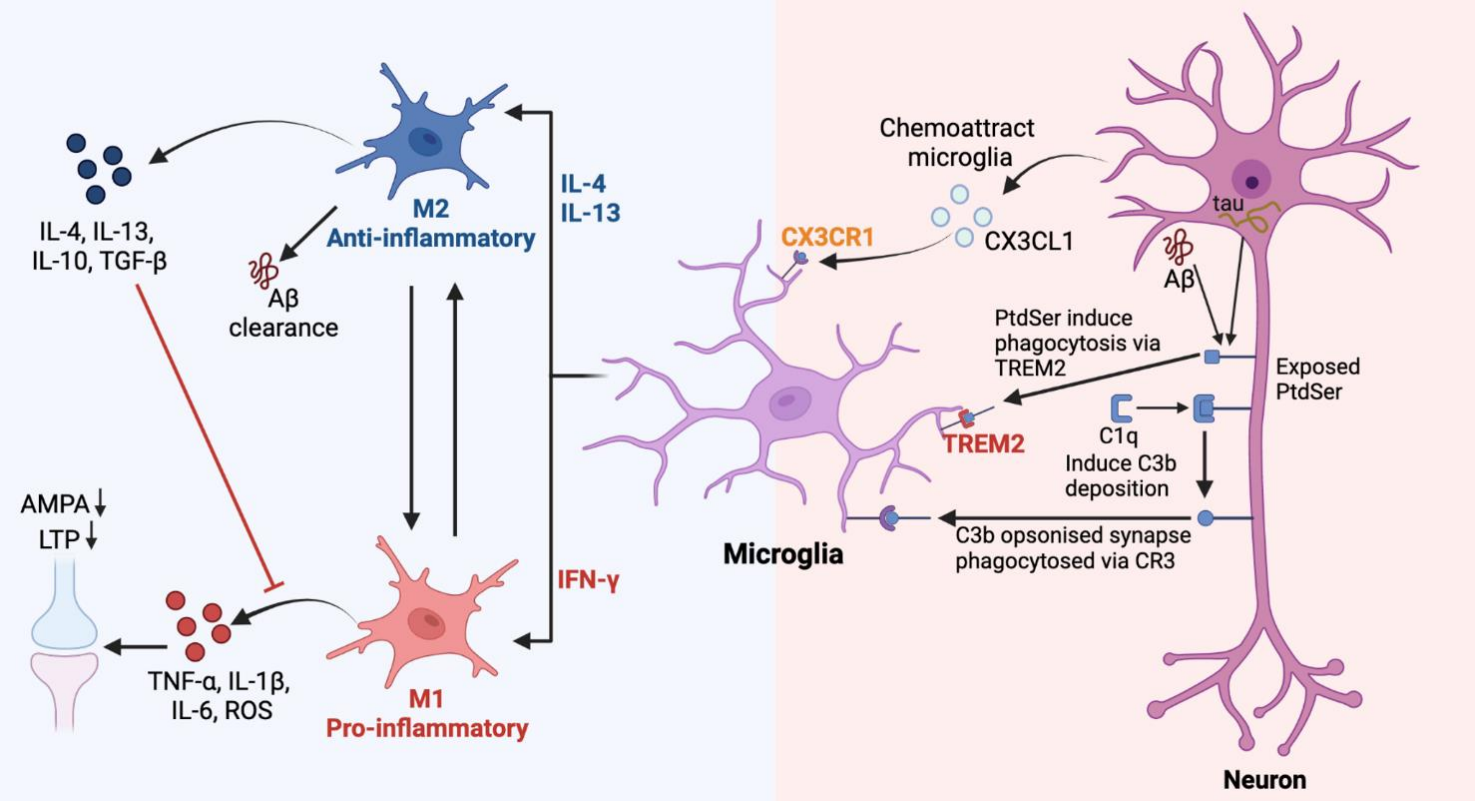
**Microglia-triggered synaptic dysfunction in AD**. Blue (left) panel: Microglia play a multifaceted role in AD through different activation phenotypes. IFN-γ as well as neuroinflammation can stimulate microglia to convert to the M1 phenotype. M1 microglia can secrete pro-inflammatory factors as well as ROS, leading to LTP impairment at synapses. M2 activation is induced by anti-inflammatory cytokines. M2 microglia can inhibit the production of pro-inflammatory factors by secreting anti-inflammatory factors and can also function as neuroprotective agents by clearing Aβ. Red (right) part: Microglia mediate synaptic loss in AD via the complement system. Aggregation of Aβ or intracellular tau proteins may cause stress on neurons, leading to the exposure of PtdSer. Exposed PtdSer can bind to complement C1q, facilitating its deposition onto neurons and inducing deposition of C3b. Opsonin C3b induces microglia to phagocytose synapses via the complement receptor C3R. In addition, neurons can also secrete CX3CL1 to chemoattract microglia via CX3CR1 receptors.

The microglia, which are macrophages that reside in the CNS, have been found to contribute to the development and progression of AD [34]. These microglial cells differentiate from progenitor cells and migrate into the CNS during early embryonic development, setting them apart from other tissue-resident macrophages [35]. Microglia play a crucial role in synaptic pruning, immune surveillance, and neuroinflammation [34, 36]. Synaptic pruning refers to the selective elimination of unnecessary or weak synaptic connections between neurons in the brain. During synaptic pruning, underutilized or less functional synapses are eliminated, while stronger and more active synapses are retained and strengthened [37]. This process helps refine and shape the neural circuitry, allowing for efficient communication between neurons. Paolicelli and Schafer et al. have confirmed that the microglia's involvement in synaptic pruning is crucial for normal brain development, optimizing neural connectivity, and promoting efficient cognitive function [36, 38]. Conversely, in AD, improper synaptic pruning occurs, resulting in abnormal neuronal connections that contribute to memory loss and cognitive decline. Two-photon imaging has revealed that "resting" microglia are highly branched and constantly monitor their microenvironment, detecting signs of injury and serving as mediators in responding to the site of injury. These resting microglia establish short-lived connections with synapses, which could be linked to microglia-mediated synaptic pruning [39]. Notably, in pathological states, the microglia remain in contact with synapses for longer periods, leading to the loss of presynaptic boutons [39]. It is speculated that this prolonged contact between the microglia and synapses may trigger synaptic loss. Recent studies have found that in AD, the microglia take up more synaptic material, supporting this speculation [40, 41]. Upon activation, the microglia initiate a series of immune processes, including phagocytosis and the release of cytokines or inflammatory mediators, to further regulate synaptic function. This section will discuss the underlying mechanisms associated with the aberrant functioning of microglia that leads to the abnormal pruning of synapses. Specifically, the mechanisms involve the complement system, cytokines, chemokines, impaired phagocytosis ability, and alterations in microglial phenotype (Fig. 1).

### 2.1 Microglia mediate synaptic loss in AD via the complement system

Studies have shown that during the development of the CNS, microglia play a crucial role in synaptic pruning, which is accomplished through the recognition of complement components C1q or C3 by microglia [36, 38, 42] (Fig. 1). Mice lacking C1q, C3, or C3R have been found to have defects in synaptic pruning [38, 42]. However, recent study suggests that the removal of synapses by microglia, which is normally confined to the developing brain, may be reactivated in aging or disease. The expression of complement components has been observed to be high during development, aging, and neurodegenerative disease, but low during adulthood, supporting the idea of reactivation later in life or in neurodegenerative disease [38, 43-45].

Similar to the peripheral immune system, classical complement proteins like C1q and C3 localize to synapses that need to be removed, serving as the "eat-me" signal for microglia expressing the C3 receptor (C3R) [38]. It has been noted that in both human and mouse aging brains, C1q levels are significantly increased and are localized around aging synapses, particularly in the hippocampus, a brain region that is highly susceptible to synaptic loss in AD [43]. Similarly, C3 has been shown to act as a mediator for synaptic loss in the mouse hippocampus during aging [46].

The complement protein C1q has been shown to bind to Aβ in human AD brain tissue, triggering the classical complement cascade. In multiple AD mouse models, including J20 and APP/PS1, soluble Aβ leads to the tagging of C1q at hippocampal synapses prior to plaque formation [47]. The absence of C1q or the use of neutralizing antibodies against C1q has been shown to prevent the synaptic loss observed in AD mouse models [47, 48]. This evidence suggests that the classical complement cascade response may play a crucial role in synaptic loss in the early stages of AD. Notably, deleting C1q does not impact Aβ levels, suggesting that C1q acts downstream of Aβ [48, 49]. Similarly, blocking C3 or knockdown of C3 has been found to prevent synaptic loss in AD and aging [46, 47, 50, 51], however, this leads to an increase in Aβ deposition [50, 52]. There are conflicting results regarding this conclusion, with some studies suggesting that C3 deficiency leads to both neurodegeneration and increased Aβ deposition [52, 53]. Additionally, studies have found that tau pathology in mouse models can cause synaptic loss, which can be prevented by blocking C1 and deleting the phagocytic complement receptor 3 (CR3) or C3 [54-56]. This indicates that tau pathology in AD may be triggered by synaptic loss through complement-mediated phagocytosis by microglia.

Microglia, with their involvement in complement-mediated synaptic pruning, may be a crucial component in the synaptic loss observed in AD (Fig. 1). Evidence has indicated that microglia are a significant source of C1q in the brain and that they mediate synaptic phagocytosis through CR3 [38, 49]. The long-term application of CSF1R inhibitors, leading to microglia depletion in APP/PS1 mice, has been shown to prevent neuronal loss and improve memory and behavioral task performance [57]. This result has been confirmed in 5xFAD mice [58]. Despite this, data from various AD mouse models including APP and 5xFAD, suggests that amyloid plaque load remains unchanged by microglia depletion [57-59]. The hypothesis that microglia depletion and blocking complement pathway activation can improve synaptic loss in AD remains controversial, but increasing evidence supports it.

### 2.2 Signals modulating microglia phagocytosis of synapses

#### 2.2.1 Find-me signals: chemokines

CX3CL1, also known as fractalkine, is a chemokine and intercellular adhesion molecule that attracts macrophages [60]. CX3CL1 is abundant and continuously expressed in CNS neurons, particularly in neurons located in the hippocampus. The signaling between CX3CL1 and CX3CR1 plays a role in various physiological processes in the brain during both development and adulthood. Studies have shown that the absence of CX3CR1 results in decreased phagocytosis of synapses by microglia during mouse development and also a reduction in the number of microglia at synapses [36, 61]. Additionally, the absence of CX3CR1 causes a decrease in microglial migration [21]. All of these findings suggest that CX3CL1 functions as a chemotactic factor that mediates the phagocytosis of synapses by microglia (Fig. 1). Notably, lacking CX3CL1 has been shown to prevent synaptic loss in the 5xTg-AD mouse model [22], suggesting that pathological activation of developmental synaptic pruning may be responsible for synaptic loss in AD.

Furthermore, Haynes et al. have found that ADP released from damaged neurons can have a chemotactic and activating effect on microglia through the activation of P2Y12 receptors [62]. The knockout of P2Y12R has been shown to reduce synaptic pruning by microglia during development [63]. However, its role in AD remains uncertain.

#### 2.2.2 Eat-me signals: phosphatidylserine

Phosphatidylserine (PtdSer) is a membrane phospholipid that serves as a "death signal" on the surface of apoptotic cells to drive synaptic pruning. Normally located in the inner leaflet of the plasma membrane, PtdSer can be exposed on the cell surface under certain conditions, such as during apoptosis, when caspase-3 activates Xkr8 to promote PtdSer externalization and subsequent engulfment of cell debris [64]. PtdSer exposure in neurons triggers microglial phagocytosis directly through receptors such as TREM2 and GPR56, or indirectly via MerTK [65-68]. Additionally, exposed PtdSer can bind to C1q, mediating microglial phagocytosis of neurons [69]. Low concentrations of Aβ in mixed neuron-glial cultures have been shown to induce PtdSer exposure on live neurons and synaptic loss in vitro [70]. Blocking or deleting PtdSer and MFG-E8 has been found to reduce Aβ-induced synaptic loss [71, 72]. Furthermore, extracellular tau has been shown to induce PtdSer exposure and microglial phagocytosis, which can be prevented through inhibition of MerTK or depletion of microglia [73] (Fig. 1). These findings suggest a potential role for PtdSer in synaptic loss in AD.

TREM2 (Triggering Receptor Expressed on Myeloid Cells 2), a receptor for PtdSer, has been found to play a significant role in AD pathogenesis in conjunction with microglia-mediated synaptic elimination. Variants of TREM2, particularly R47H, have been identified through genome-wide association studies as being strongly associated with AD [74, 75]. Gene deletion of TREM2 has also been found to reduce synaptic pruning in developing mice [76] (Fig. 1). However, the introduction of the R47H variant into the PS19AD mouse model decreased synaptic loss [77]. In human AD brain tissue carrying the R47H variant of TREM2, a decrease of synaptic markers in microglial phagolysosomes was also observed [77]. This contradicts previous human genome studies [74, 75], suggesting that the effect of TREM2 on synaptic loss may depend on the stage of disease development in AD.

#### 2.2.3 Don't-eat-me signals: CD47

CD47 is a member of the immunoglobulin superfamily and is commonly found on the surface of most mammalian cell membranes. It functions as a "don't eat me" signal that hinders synaptic pruning, thus preserving active synapses from being engulfed. CD47 is expressed on synapses during development, and its receptor SIRPα is highly expressed on microglia at the peak of pruning periods in development, preventing microglia-mediated synaptic phagocytosis [78]. Interestingly, CD47 and CD36 play a role in the phagocytosis of fibrillar Aβ by microglia [79]. Although there is evidence that suggests CD47's involvement in AD pathogenesis, further research is needed to determine if it plays a role in microglia-mediated synaptic phagocytosis in AD.

### 2.3 Cytokines involved in microglia-synapse interaction

#### 2.3.1 Microglia regulate synaptic plasticity via the secretion of cytokines

Microglia, the primary immune cells of the central nervous system, can affect synaptic function and plasticity through the secretion of pro-inflammatory cytokines, such as IL-1β, TNF-α, and IL-6, as well as ROS and BDNF [28]. Changes in synaptic plasticity, including long-term potentiation (LTP) and long-term depression (LTD), may occur before microglia-mediated synaptic pruning in AD. Inflammatory triggers, like LPS, activate NADPH oxidase in microglia, causing the release of ROS. This, in turn, activates protein phosphatase 2A (PP2A) in neurons and results in LTD due to GluA2-dependent AMPA receptor endocytosis [80]. Aβ, a hallmark of AD, also increases ROS production in microglia and may contribute to synaptic dysfunction [81] (Fig. 1).

The pro-inflammatory cytokines such as IL-1β and TNF-α are increased in AD [23]. Previous studies have demonstrated that these pro-inflammatory factors can affect synaptic LTP (Fig. 1). For example, CX3CR1 knock-out mice showed increased levels of IL-1β, cognitive impairment, and reduced LTP [24]. Infusing IL-1β receptor antagonists reversed these effects, suggesting that high levels of IL-1β may be linked to synaptic dysfunction in AD [24]. On the other hand, the impact of TNF-α on LTP is controversial, with some studies indicating inhibition of LTP in the hippocampus [82, 83], while others suggest facilitation of LTP [84, 85]. This discrepancy may be due to differences in experimental methods, such as timing of TNF-α application and monitoring. Pettigrew et al. propose that overexpression of the TNF-α gene may lead to LTP deficits if synaptic changes are monitored for an extended period [85]. Additionally, IL-1β and TNF-α can regulate all major classes of synaptic voltage-gated channels [86].

#### 2.3.2 Cytokines regulating microglial phagocytosis of synapses

A variety of cytokines have been found to regulate microglia phagocytosis. TNF-α, a major pro-inflammatory cytokine, is elevated in the brain and plasma of AD patients. Prolonged TNF-α overexpression in 3xTg-AD mice results in increased AD-related neuropathology and eventually neuronal death [87, 88]. A study found that TNF-α prompts microglia to phagocytose live neurons, leading to neuronal loss in mixed glial-neuronal cultures, which can be prevented by inhibiting microglia phagocytosis [25]. Another cytokine that stimulates microglia phagocytosis is IL-33, a member of the interleukin-1 cytokine family primarily produced by astrocytes [89, 90]. IL-33 activates microglia through the ST2 receptor and the NF-κB, p38, JNK, and ERK pathways [89]. Reduced microglia-mediated synaptic pruning was observed in IL-33 knockout mice during developmental stages [90]. IL-33 also triggered a transition in microglia status and increased phagocytosis of Aβ plaques in APP/PS1 mice [91, 92]. Other cytokines, such as IL-4 and IFN-β, may impact microglia phagocytosis by inducing different activation phenotypes [29, 93] (Fig. 1).

### 2.4 Microglia state and synaptic function

The traditional classification of microglia phenotype involves dividing it into classical (M1) and alternative (M2) states. M1 activation is a pro-inflammatory and neurotoxic state that is typically triggered by the TLR and IFN-γ signaling pathways through the influence of IFN-γ and LPS [94]. M1 microglia are known to produce pro-inflammatory factors such as TNF-α, IL-1β, and IL-6 [95]. Additionally, they express NADPH oxidase, which generates superoxide, ROS, and has a close association with the neurotoxicity of amyloid precursor proteins and changes in synaptic plasticity [80, 96]. M1 polarization was found to inhibit fibrillar Aβ-stimulated phagocytosis, which may impact the clearance of Aβ by microglia in AD [97] (Fig. 1). On the other hand, M2 activation is induced by anti-inflammatory cytokines such as IL-4 and IL-13 [94] (Fig. 1). M2 microglia are a range of different activation phenotypes that can be further categorized into M2a, M2b, and M2c. These subtypes have overlapping but distinct biochemical signatures and activation pathways. M2 microglia possess both anti-inflammatory and reparative functions, counteracting inflammatory responses by secreting anti-inflammatory factors such as IL-4, IL-13, IL-10, and TGF-β [98] (Fig. 1). IL-4 and IL-10 curb the production of pro-inflammatory factors such as IL-6 and TNF-α and decrease NO release [99, 100]. Microglia located around Aβ plaques have been found to exhibit an M2 phenotype, as indicated by Ym1, which reflects the fact that microglia can exert neuroprotective effects by degrading Aβ plaques [101]. The activation of microglia is dynamic, and their phenotype is continually changing. Fan et al. observed two microglia activation peaks in AD: an early protective peak and a later pro-inflammatory peak, highlighting the dual role of microglia activation in the pathogenesis of AD [102].

The brain microglia from AD mouse models (APP/PS1 and 5xFAD) and aging have been studied using single-cell RNA sequencing and the results have shown that there is a dynamic, context-dependent microglia type known as disease-associated microglia (DAM) [103, 104]. The DAM in 5xFAD AD mice is characterized by the upregulation of genes related to phagocytosis, such as Axl, Clec7a, TREM2, and ApoE [103]. As previously mentioned, TREM2 helps microglia engulf cells exposed to PtdSer and is closely linked to AD. ApoE helps with the phagocytosis of apoptotic cells by microglia [105], and Atagi et al. have found that it can bind to microglial TREM2 to enhance the phagocytosis of apoptotic neurons [106]. This suggests that the upregulation of phagocytosis-related genes in DAM may enhance its phagocytic ability.

Moreover, Allendorf et al. have shown that fibrillar Aβ and Tau can increase the surface sialidase activity and desialylation of microglia [107]. Desialylation of microglia increases its phagocytosis, which is activated by CR3 [107]. Adding Aβ to glial-neuronal cultures leads to neuronal loss, which can be prevented by using sialidase inhibitors or blocking CR3 [107]. This implies that neuroinflammation in AD may cause desialylation of microglia, leading to increased phagocytosis of healthy neurons.

## 3. Astrocyte and synaptic dysfunction

Astrocytes, the largest type of glial cells in the central nervous system, play a crucial role in supporting and separating neurons and contribute to the formation of the blood-brain barrier. They are involved in the envelopment of synapses and can help sustain these connections [90, 108]. Astrocytes play a pivotal role in the maintenance and plasticity of synapses through their formation of a specialized synaptic structure referred to as the "tripartite synapse". This structure encompasses both pre- and post-synaptic elements, enabling astrocytes to actively regulate neurotransmitters while also offering crucial support for energy metabolism [109-112]. In addition, astrocytes have phagocytic and clearance abilities similar to those of microglia [113]. Recent studies suggest a close relationship between astrocytes and AD, which can result in impairments in learning and memory [114-116]. In cases of harmful inflammatory cytokine attacks and abnormal accumulations of metabolites, astrocytes may enter into a state of reactive astrogliosis [117, 118], which may impact synaptic plasticity [118]. Studies have shown that astrocytes significantly alter synaptic plasticity in AD patients in various ways [119]. Three main mechanisms involved in this process are the secretion and clearance of neurotransmitters, regulation of ion concentrations, and neuroinflammation.

### 3.1 Secretion and clearance of neurotransmitters

Synaptic transmissions can be divided into two categories: excitatory and inhibitory. Excitatory synaptic transmissions are mainly driven by glutamatergic synapses and are activated by AMPA and NMDA receptors, leading to local depolarization. Inhibitory synaptic transmissions activate GABA and glycinergic signaling. The balance between excitatory and inhibitory transmissions is crucial in maintaining synapse plasticity, and an imbalance can lead to central neurodegenerative diseases such as AD [120]. The glutamatergic/ GABAergic balance is a critical factor in information transfer, with too many excitatory transmitters causing contamination and inhibitory transmitters suppressing information transmission [119]. Studies have shown that this balance is commonly impaired in AD patients [26, 121]. AD mice have also been found to be susceptible to excitatory/inhibitory dysfunction in the prefrontal cortex [122].

**Figure 2.**
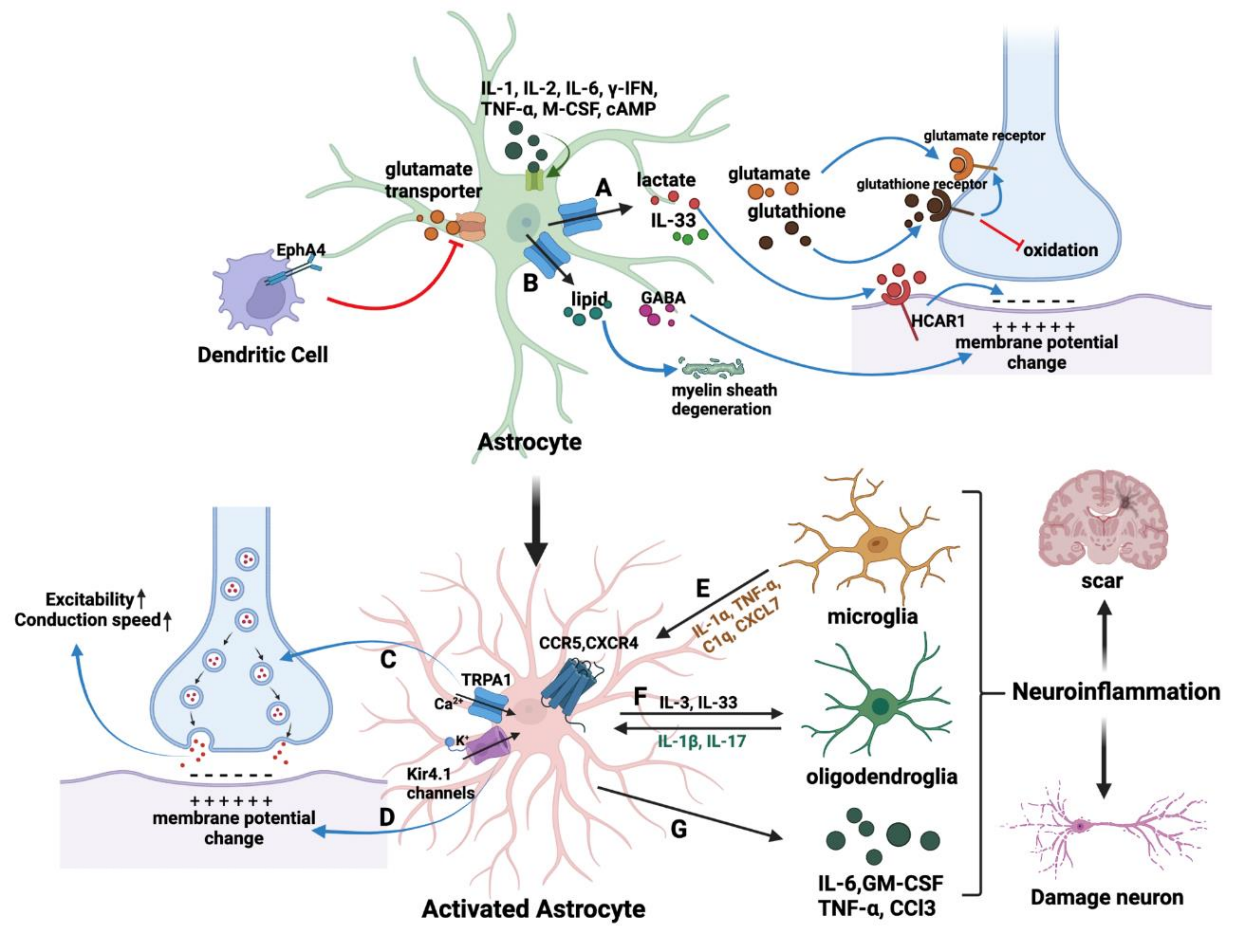
**Astrocyte-elicited synaptic dysfunction during AD**. **(A)** Astrocytes secrete traffic dissociative energy substances such as lactate, D-serine, and glutamate to strengthen the interaction between synapses. **(B)** Astrocytes produce inhibitory neurotransmitters such as GABA to induce negative reactions and saturated lipids containing APOE and APOJ lipoparticles to poison neurons. **(C)** The non-selective calcium channel TRPA1 on astrocytes can increase intracellular Ca+ and release ATP to stimulate the excitability and conduction velocity of neurons. **(D)** α2-NKA and Kir4.1 channels regulate synaptic membrane potential by affecting K+ concentration. **(E)** Interaction between astrocytes and microglia: IL-1α, TNF-α, C1q and CXCL7 from active microglia induce A1 astrocyte-stimulated neuron and oligodendrocyte death. Microglia activate CXCR4 and CCR5 on astrocytes to stimulate synapse-damaging glutamate release. **(F)** Interaction between astrocytes and oligodendrocytes. IL-1β and IL-17 from oligodendrocytes compete with astrocytes at synapses, exacerbating inflammation and blocking normal function of nervous system integrity. **(G)** Astrocytes directly secrete IL-6, GM-CSF, TNF-α, and CCL3 (MIP-1α) to stimulate the NF-κB and PI3K-Akt signaling pathways.

Astrocytes have a critical function in modulating both excitatory and inhibitory synaptic transmissions. During reactive astrogliosis, changes in the morphology of the astrocytes, such as an increase in soma size and dendrite thickness, can cause tangles between synapses and interrupt synapse trafficking and secretion [109, 123] (Fig. 2). Cytokines such as IFN-γ, IL-1, IL-2, IL-6, TNF-α, and M-CSF can evoke astrogliosis. Increased cAMP concentrations also allow astrocytes to traffic energy substances, such as lactate, D-serine, glutamate, and other gliotransmitters, via gap junctions to strengthen synapse interactions [117, 124, 125]. Astrocytes can also synthesize IL-33 to increase the number of excitatory synapses when neuronal activities are blocked in the hippocampus [126]. Additionally, astrocytes secrete several neurotrophic factors, including BDNF, VEGF, and TGF-β, to restore or protect synapses and induce the reconstruction of brain vessels in injured areas [27].

The glucose metabolism of astrocytes also plays a role in regulating synaptic plasticity (Fig. 2). Astrocytes can use glucose or glycogen to produce extracellular L-lactate, D-lactate, and glutathione [127]. These substances can bind to the HCAR1 receptor in post-synaptic membranes to adjust synaptic plasticity. Glutathione can also prevent synapses from oxidation and activate glutamate receptors [127]. In addition to inducing positive responses, astrocytes can also secrete inhibitory neurotransmitters, such as GABA, to induce negative responses [128] (Fig. 2). Lipid mechanisms have also been found to play a role in synapse degeneration, with astrocytes producing saturated lipids containing ApoE and ApoJ to induce neuronal death [129] (Fig. 2).

The normal functioning of synapses relies on neurotransmitters. An imbalance in neurotransmitter levels can lead to their accumulation and toxicity, causing irreversible damage to synapses [130]. Excessive extracellular glutamate levels can lead to neuronal cell death through excitotoxicity. The uptake of glutamate is primarily mediated by the sodium-dependent symporter GLT-1, which is predominantly expressed in astrocytes [131]. A previous study reported a decreased expression of GLT-1 mRNA in the hippocampus of individuals with AD [132]. Overexpression of GLT-1 in APP Swedish mice has been shown to reduce extracellular glutamate concentration and ameliorate cognitive deficits [133, 134]. Hence, astrocytes engage in the regular clearance of extracellular neurotransmitters in order to uphold the appropriate functioning and development of synapses. If the clearance is not sufficient, the accumulation of excitatory neurotransmitters like glutamate can result in excitatory toxicity and harm to synapses. Overactive astrocytes exacerbate the pathological progression of AD due to imbalanced synaptic transmission.

### 3.2 Regulation of ionic concentration

Astrocytes regulate neurotransmission and synaptic plasticity through their Ca^2+^ signaling and gliotransmitter release. TRPA1, a non-selective calcium channel on astrocytes, can increase intracellular Ca^2+^ levels and release ATP, leading to an increase in neuronal excitability and conduction velocity [135, 136]. Additionally, astrocytes express Gq GPCRs, which can be activated by neurotransmitters released from presynaptic terminals, resulting in an increase of Ca^2+^ in astrocytes [137]. The elevated Ca^2+^ levels in astrocytes, triggered by Gq GPCR activation, can result in the release of gliotransmitters and affect nearby synapses [137] (Fig. 2).

The enhancement of synaptic transmission over time, known as LTP, forms the foundation of learning and memory and is a widely studied aspect of synaptic plasticity [138]. Jh et al. have shown that astrocyte inositol triphosphate receptor type 2 (IP3R2) - dependent calcium signaling plays a crucial role in the late-phase of LTP [139]. Popov et al. discovered that deficiencies in the astrocyte's ability to reorganize calcium events can result in reduced LTP using patch-clamp and calcium imaging [140].

Astrocytes have the ability to regulate synaptic membrane potential through controlling the concentration of potassium (K^+^) [111]. A study by Mann et al. found that increased levels of α2-Na/K adenosine triphosphatase (α2-NKA) in astrocytes were present in AD and that reducing α2-NKA prevented the buildup of tau [141] (Fig. 2). Photostimulation on astrocytes decreases the resting potassium concentration, thereby increasing the likelihood of action potentials at synapses [142]. Additionally, inwardly rectifying Kir4.1 channels in astrocytes, linked to cerebral disorders, transmit surplus potassium to intercellular spaces [143]. In summary, calcium and potassium activities in astrocytic processes are generated by synapses, and astrocytes can modulate neuronal activity in both directions through the release and clearance of neuroactive chemicals dependent on calcium or potassium.

### 3.3 Neuroinflammation

The differentiation of astrocytes into two different subtypes plays a role in inflammation and impacts synaptic plasticity [112]. The inflammatory type, also known as A1, disrupts synaptic integrity, and is implicated in various neurodegenerative diseases [144]. In contrast, the A2 phenotype provides neuroprotection and suppresses inflammation in astrocytes by intercepting the AKT/STAT3 signaling pathway [145, 146]. At the early stages of AD pathology, astrocyte-mediated inflammation can help remove abnormal cells and substances such as Aβ, but if this process becomes uncontrolled, excessive inflammation can harm surrounding cells and affect synaptic function.

IL-1α, TNF, C1q, and Aβ can trigger A1 astrocytic activation by downregulating the NF-κB signaling pathway and upregulating the PI3K-Akt pathway [147]. Upon activation, astrocytes can either directly secrete cytokines for neuroinflammation or attract other glial cells to modify the synapse plasticity coefficient [148]. Excessive inflammation may lead to neuronal degeneration and synapse loss, thereby contributing to AD development (Fig. 2).

Just like microglia, astrocytes have the ability to secrete cytokines and chemokines such as IL-6, GM-CSF, TNF-α, and CCL3 (MIP-1α), which are directly targeted by the transcription factor NF-κB [149]. The level of IL-6 is significantly elevated in AD, which enhances the trafficking of glutamate and NMDA receptors in synapses and leads to neuronal loss [150] (Fig. 2). It is believed that IL-6 may disrupt synaptic plasticity during AD by damaging synapses and inducing excitotoxicity. Furthermore, CCL3 impairs synaptic recovery and plasticity after secondary injury in the central nervous system [151].

## 4. Oligodendrocytes and synaptic dysfunction

**Figure 3.**
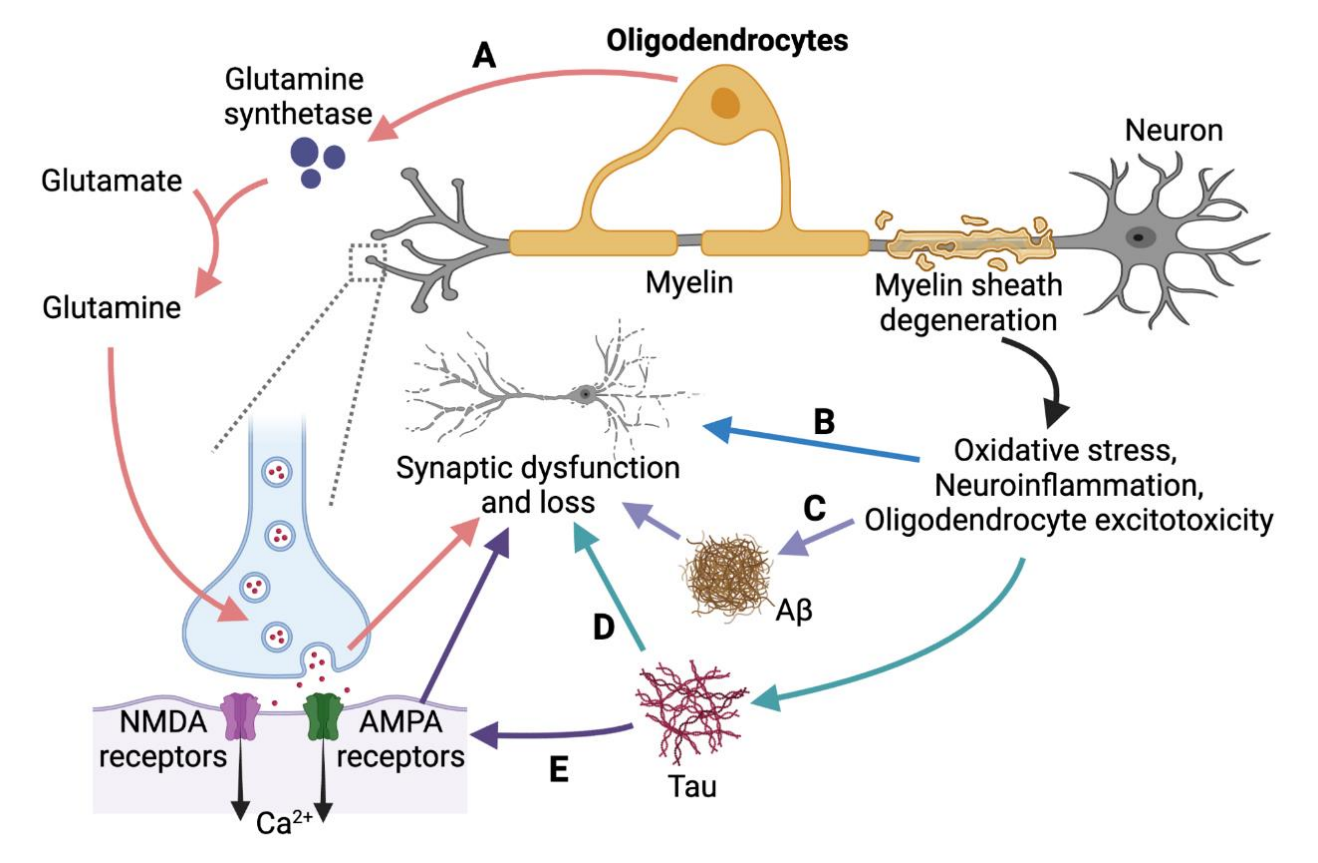
**Oligodendrocyte-induced synaptic dysfunction in AD**. **(A)** Abnormal expression of glutamine synthetase by oligodendrocytes contributes to the progression of AD by disrupting the regulation of neurotransmitter cycling and release. **(B)** The loss of myelin sheath is associated with oxidative stress, neuroinflammation, and oligodendrocyte excitotoxicity, leading to synaptic dysfunction and memory impairment, and may even precede the formation of (C) amyloid and (D) tau pathology. **(E)** The tau protein disrupts the anchoring and function of AMPARs and NMDARs, resulting in abnormal synaptic transmission.

The role of oligodendrocytes in the CNS is to create a protective myelin sheath around axons, support the normal function of neurons, and regulate neurotransmission [152]. The myelin insulation provided by oligodendrocytes offers metabolic and nutritional support for the axons, and it plays a critical role in maintaining neuronal homeostasis and facilitating motor skill learning [153, 154]. The creation of new myelin segments is an important form of plasticity that helps to modify the properties of CNS circuits [155]. Oligodendrocytes contribute to the enhancement of action potential conduction velocity by producing myelin, consequently impacting synaptic transmission [156]. Demyelination, characterized by the loss of myelin surrounding axons, has been observed as a prominent feature during the progression of AD. This demyelination process contributes to impaired neuronal signaling, disrupting the efficient transmission of electrical impulses between neurons. Furthermore, the impairment in the processes involved in myelin repair exacerbates the degeneration of axons, ultimately leading to the demise of neurons (Fig. 3). Declining myelination and memory deficits go hand-in-hand in aging, and the loss of the myelin sheath might be the earliest step in the development of AD, preceding the formation of amyloid and tau pathology [157] (Fig. 3). The exact molecular mechanisms involved are not yet clear, but they are thought to be related to oxidative stress, neuroinflammation, oligodendrocyte excitotoxicity, and the accumulation of Aβ peptides [158, 159] (Fig. 3). Previous studies have shown that experimentally inducing damage to myelin leads to hyperphosphorylation of tau, which disrupts the anchoring and function of AMPARs and NMDARs and results in abnormal synaptic transmission [160, 161] (Fig. 3). Additionally, inhibiting synaptic activity exacerbates the accumulation of tau oligomers in swollen lysosomes and further induces synaptic degeneration [162]. However, Wang et al. found that boosting myelination rescued synapse loss and improved memory function in aging mice [163].

Furthermore, oligodendrocytes secrete growth factors and signaling molecules, such as BDNF and insulin-like growth factor-1 (IGF-1), that regulate neurotransmitter release from presynaptic terminals, thereby influencing synaptic plasticity [164, 165]. Moreover, oligodendrocytes regulate neurotransmitter cycling and release by expressing glutamine synthetase, which converts glutamate to glutamine [166-168]. Previous studies have indicated that disruptions in glutamate regulation lead to severe neuropathology [169]. Hence, changes in the expression and activity of glutamine synthetase caused by abnormal oligodendrocyte function may also contribute to AD [170] (Fig. 3).

## 5. Oligodendrocyte precursor cells and synaptic dysfunction

**Figure 4.**
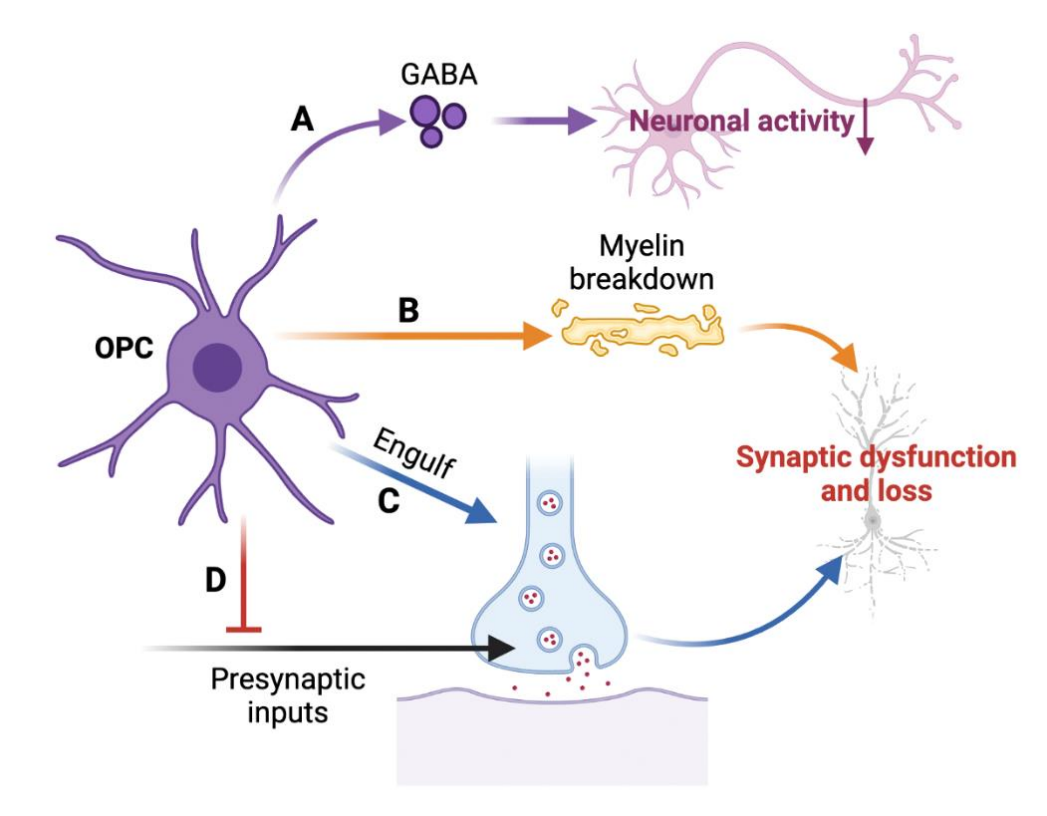
**Oligodendrocyte precursor cell-promoted synaptic dysfunction in AD**. **(A)** Oligodendrocyte precursor cells (OPCs) regulate inhibitory neuronal activity by secreting the neurotransmitter GABA. **(B)** Impaired OPCs cause myelin breakdown leading to synaptic dysfunction and the development of AD pathology. **(C)** OPCs control the number of synapses by engulfing and phagocytosing them, however, this process is altered in AD. **(D)** The capacity of OPCs to modify the number of synapses through the engulfment of presynaptic inputs is lost during AD.

The function of oligodendrocyte precursor cells (OPCs), also known as NG2 glial cells, in the CNS is to modulate neuronal synaptic activity. OPCs play a crucial role in the process of remyelination and synapse repair. OPCs have the ability to differentiate into mature oligodendrocytes, which are responsible for the myelination process [171, 172]. The remyelination process aids in the restoration of normal neuronal signaling and facilitates functional recovery. Bergles et al. used electron microscopy to show that vesicle-filled axon terminals form synaptic connections with OPCs in the young and adult hippocampus [173]. These synapses between OPCs and neurons provide the necessary conditions for supporting myelination through indirect communication [174]. Furthermore, axon-OPC synapses regulate the myelination behavior of oligodendrocyte processes directly, similar to how neurons regulate them [175]. Myelin breakdown, which occurs when the repair function of OPCs is impaired, is believed to be the initial step in AD pathology [176-178] (Fig. 4). Myelin remodeling is essential for memory encoding [154, 179, 180]. In both gray and white matter, OPCs play a crucial role in regenerating pathologically lost myelin and providing new myelin by producing remyelinating oligodendrocytes [181-184]. Therefore, disrupting OPCs negatively affects myelin replacement and leads to synaptic dysfunction in AD [185] (Fig. 4).

Moreover, OPCs establish interactions with neurons and synapses, offering nutritional support and releasing signaling molecules that facilitate synaptic remodeling and repair processes.

OPCs can regulate inhibitory neuronal activity by releasing GABA [178, 186] (Fig. 4). A study by Zhu et al. showed that removing OPCs from the prefrontal cortex negatively impacted glutamate receptor trafficking and glutamatergic neurotransmission [184]. Auguste et al. discovered that, compared to mature oligodendrocytes, OPCs have the ability to affect the number of synapses by engulfing presynaptic inputs [187] (Fig. 4). Additionally, OPCs have the potential to contribute to the formation of short-term memory by modulating synaptic transmission or engaging in synapse engulfment [188]. However, it is important to note that this function is disrupted in AD (Fig. 4).

Through their ability to facilitate the repair of impaired synapses, OPCs contribute to the restoration of regular neuronal communication and the preservation of cognitive function. Emerging studies indicate a potential involvement of OPCs in the pathology of AD [181-184, 187]. Dysfunction or depletion of OPCs can interfere with the synaptic repair mechanism and contribute to the observed synaptic degeneration in AD. Augmenting the function of OPCs and promoting synaptic repair hold promise as potential therapeutic strategies for AD.

## 6. Therapeutic approaches targeting glial cells in clinical trials for AD

Currently, the treatment approaches for AD encompass both pharmacological interventions and non-pharmacological strategies. In recent years, there has been a growing focus on developing drugs targeting inflammatory pathways and glial cells, which have shown promising therapeutic effects in animal studies and have received approval for clinical research, primarily in phase I/II clinical trials. As of 2023, a total of 24 drugs specifically addressing neuroinflammation have entered the preclinical research phase, constituting approximately 7% of all drugs undergoing clinical trials [189]. One notable disease-modifying therapy drug that targets neuroinflammation, currently in phase III clinical trial (National Clinical Trial number NCT04669028), is NE3107. This small molecule has exhibited neuroprotective effects in AD mouse models [190]. NE3107 selectively inhibits ERK- and NF-κB-mediated inflammatory signaling pathways while down-regulating various inflammatory mediators such as C1q, IL-6, and TNF-α [191, 192]. Its efficacy in improving cognitive decline and slowing disease progression in patients with mild to moderate AD is being investigated through clinical trials employing volumetric magnetic resonance imaging and 18F-fluorodeoxyglucose positron emission tomography-computed tomography (18F-FDG PET-CT) [190].

In this review, we provided a detailed explanation of the role and mechanisms underlying synaptic dysfunction and loss mediated by different types of glial cells in AD. Potential therapeutic avenues targeting relevant receptors and inflammatory responses are discussed. While preclinical studies have identified promising strategies for AD treatment, clinical trials are yet to be conducted. For instance, ibuprofen, a commonly used nonsteroidal anti-inflammatory drug, has demonstrated the ability to reduce the release of pro-inflammatory factors from microglial cells, decrease Aβ plaques, and slow down disease progression in animal models [193]. Inhibitors targeting the P2X7 receptor have shown efficacy in mitigating the inflammatory response of microglial cells [194]. Targeting immune receptors on microglial cells, such as TREM2 or CD33, holds potential therapeutic value by enhancing phagocytic activity against Aβ [195, 196]. Stem cell therapy targeting glial cells has also shown promise in ameliorating memory impairment in AD mice. Transplantation of mesenchymal stem cells has been shown to regulate the activation status of astrocytes [197] and microglia [198] by modulating the secretion of immune cell factors and growth factors, thereby reducing inflammation and Aβ plaques. Fluoxetine has exhibited cognitive improvement by inhibiting astrocyte activation [199]. NaBP, by elevating the levels of neurotrophic proteins and brain-derived neurotrophic factor (BDNF) in astrocytes, exerts neuroprotection and improves cognitive impairments [200]. However, the complex pathological conditions observed in AD patients have resulted in some drugs that showed promising results in preclinical studies not achieving the anticipated effects in clinical trials. Therefore, it is imperative to conduct further investigations into the synaptic dysfunction regulated by glial cells in AD to unveil additional potential treatment strategies.

**Table 1:** The impact of different molecules on the synaptic dysfunction induced by glial cells in Alzheimer's disease.

Molecules	Produced by or impacting glia	The effects in AD	References
IL-1β	Impact microglia	Increased IL-1β level reduces cognitive impairment and LTP	[23, 24]
IL-4, IL-13	Impact microglia	Induce M2 activation and impact microglia phagocytosis	[29, 94, 98]
IL-4, IL-10	Impact microglia	Downregulate the production of pro-inflammatory factors (IL-6 and TNF-α)	[99, 100]
IL-33	Secreted by astrocyte	Alteration microglia status and increase phagocytosis of Aβ plaques	[90, 92]
TNF-α	Impact microglia	Prompt microglia to phagocytoses live neurons and thus cause neuronal loss. However, the effect on LTP is controversial	[25]; [82-85]
IFN-β	Impact microglia	Induce different activation states and impact microglia phagocytosis	[93]
C1q	Impact microglia and astrocyte	Increase microglial phagocytosis and synaptic loss; trigger A1 astrocytic activation and secrect neuroinflammation cytokines	[47, 48]; [202]
C3	Impact microglia	Conflict effects: blocking C3 prevent synaptic loss caused by Tau pathology and increases Aβ	[54, 56]; [50, 52]
CX3XL1	Impact microglia	Positive for microglial migration and synaptic loss	[21, 22]
P2Y12	Impact microglia	P2Y12R knockout reduce synaptic pruning	[63]
TREM2	Microglial TREM2 (PtdSer receptor)	Relevant to synaptic pruning; Positive cause Aβ-induced synaptic loss	[76];[71]
Fibrillar Aβ	Impact microglia	Induce desialylation of microglia, and leading to neuronal loss	[107]
L- or D-lactate	Produced by astrocyte	Bind to post-synaptic membrane and adjust synaptic plasticity	[127]
GABA	Released by astrocyte or OPC	Regulate inhibitory neuronal activity to neuron	[128, 178, 186]

### Conclusion and perspective

The functional impact of synaptic dysfunction in AD can be severe, as glial cells play a vital role in affecting neurons and brain development. In this review, we highlight the evidence and role of various types of glial cells, including microglia (Fig. 1), astrocytes (Fig. 2), oligodendrocytes (Fig. 3), and oligodendrocyte precursor cells (Fig. 4) in causing synaptic dysfunction in the progression of AD. And we compiled Table 1 to summarize various representative molecules that are relevant to glia-induced synaptic dysfunction during the progression of AD. From this, we can infer that microglia release pro-inflammatory substances and fail to maintain glutamate homeostasis, leading to synaptic dysfunction in AD. Additionally, the synaptic buildup of complement system proteins results in glial-mediated synaptic phagocytosis and loss. Astrocytes impair synaptic plasticity by controlling ion concentrations. Besides, the interaction between astrocytes, microglia, and oligodendrocytes also lead to synaptic dysregulation: activated microglia induces the release of IL-1α, TNF-α, and C1q, causing the formation of astrocyte A1 phenotype, which leads to the death of neurons and oligodendrocytes [201, 202]. Microglia activate CXCR4 and CCR5 on astrocytes to stimulate the release of synaptic damaging glutamate [203]. Microglia can also synthesize CXCL7 to harm astrocytes and myelin [204], leading to synaptic dysfunction. The interaction of microglia and astrocytes has a more negative effect than individual cells [205].

Recent advancements in *in vitro* models and techniques have enabled significant progress in investigating the interactions between glial cells and neurons. However, most research in this area still relies on primary cultured cells from animal models, despite substantial disparities between animal and human neurons and glial cells. This is particularly important to note as human neurons and glial cells exhibit distinct protein expressions and activities that are relevant to the pathology and dynamics of AD. In recent years, there has been a shift towards utilizing human induced pluripotent stem cells (iPSCs) to generate neurons and glial cells for AD research. iPSC induction and subsequent differentiation have emerged as indispensable tools for unraveling the underlying mechanisms of AD. However, the differentiation and culturing procedures for human iPSCs are intricate, and the induction of iPSC-derived neurons and glial cells is time-consuming and lacks experimental reproducibility. Thus, further advancements are necessary to refine the construction of in vitro models.

A recent study conducted by Bassil et al. demonstrated the successful construction of an AD model using human iPSC-derived neurons, microglia, and astrocytes, which recapitulated crucial aspects of AD progression, including Aβ plaque formation, synaptic loss, neuroinflammatory responses, and neuronal death [206]. Leveraging *in vitro* constructed human AD models as research tools offers tremendous potential for enhancing our comprehensive understanding of the disease mechanism and expediting drug development efforts. Moreover, it is conceivable that such models may become pivotal methodologies in investigating the intricate mechanisms underlying AD in the future.

Recent advances in cell biology techniques have highlighted the contribution of glial cells to synaptic dysfunction in AD, emphasizing the need for further investigation into the underlying mechanisms and signaling pathways involved. While microglia and astrocytes have been extensively studied in neuroinflammation, the contribution of OPCs and oligodendrocytes to synaptic dysfunction in AD should not be overlooked. Despite their potential importance in the context of synaptic dysfunction in AD, the involvement of OPCs and oligodendrocytes remains less understood.

To develop effective therapies targeting synaptic alterations, it is imperative to conduct further investigations on glia-induced synaptic dysfunction under both physiological and pathological conditions. Such studies have the potential to identify novel therapeutic targets for AD and offer promise for advancing our understanding of the disease. We hope to inspire more investigators to focus on this aspect of AD progression, as it represents a fertile area for future scientific exploration.
